# The integrin αvβ6 drives pancreatic cancer through diverse mechanisms and represents an effective target for therapy

**DOI:** 10.1002/path.5320

**Published:** 2019-07-30

**Authors:** Claire S Reader, Sabari Vallath, Colin W Steele, Syed Haider, Adam Brentnall, Ami Desai, Kate M Moore, Nigel B Jamieson, David Chang, Peter Bailey, Aldo Scarpa, Rita Lawlor, Claude Chelala, Stephen M Keyse, Andrew Biankin, Jennifer P Morton, TR Jeffry Evans, Simon T Barry, Owen J Sansom, Hemant M Kocher, John F Marshall

**Affiliations:** ^1^ Centre for Tumour Biology, Barts Cancer Institute, CRUK Centre of Excellence Queen Mary University of London, John Vane Science Centre London UK; ^2^ Cancer Research UK Beatson Institute Glasgow UK; ^3^ Institute of Cancer Research London UK; ^4^ Centre for Cancer Prevention, Wolfson Institute of Preventive Medicine Queen Mary University of London London UK; ^5^ Academic Unit of Surgery, School of Medicine, College of Medical, Veterinary and Life Sciences University of Glasgow, Glasgow Royal Infirmary Glasgow UK; ^6^ West of Scotland Pancreatic Unit Glasgow Royal Infirmary Glasgow UK; ^7^ Wolfson Wohl Cancer Research Centre, Institute of Cancer Sciences University of Glasgow Glasgow UK; ^8^ ARC‐NET Research Centre for Applied Research on Cancer University of Verona Verona Italy; ^9^ Centre for Molecular Oncology, Barts Cancer Institute, CRUK Centre of Excellence Queen Mary University of London, John Vane Science Centre London UK; ^10^ Division of Cancer Research, University of Dundee, James Arrott Drive Ninewells Hospital and Medical School Dundee UK; ^11^ Bioscience, Oncology R&D, AstraZeneca Cambridge UK

**Keywords:** PDAC, integrin, 264RAD, αvβ6, pancreas, cancer, transgenic, mouse model

## Abstract

Pancreatic ductal adenocarcinoma (PDAC) has a 5‐year survival rate of less than 4% and desperately needs novel effective therapeutics. Integrin αvβ6 has been linked with poor prognosis in cancer but its potential as a target in PDAC remains unclear. We report that transcriptional expression analysis revealed that high levels of β6 mRNA correlated strongly with significantly poorer survival (*n* = 491 cases, *p* = 3.17 × 10^−8^). In two separate cohorts, we showed that over 80% of PDACs expressed αvβ6 protein and that paired metastases retained αvβ6 expression. *In vitro,* integrin αvβ6 promoted PDAC cell growth, survival, migration, and invasion. Treatment of both αvβ6‐positive human PDAC xenografts and transgenic mice bearing αvβ6‐positive PDAC with the αvβ6 blocking antibody 264RAD, combined with gemcitabine, significantly reduced tumour growth (*p* < 0.0001) and increased survival (log‐rank test, *p* < 0.05). Antibody therapy was associated with suppression of tumour cell activity (suppression of pErk growth signals, increased apoptosis seen as activated caspase‐3) and suppression of the pro‐tumourigenic microenvironment (suppression of TGFβ signalling, fewer αSMA‐positive myofibroblasts, decreased blood vessel density). These data show that αvβ6 promotes PDAC growth through both tumour cell and tumour microenvironment mechanisms and represents a valuable target for PDAC therapy. © 2019 The Authors. *The Journal of Pathology* published by John Wiley & Sons Ltd on behalf of Pathological Society of Great Britain and Ireland.

## Introduction

Pancreatic ductal adenocarcinoma (PDAC) is the fifth leading cause of cancer‐related death with a 5‐year survival rate of less than 4% 1 largely due to the asymptomatic nature and late‐stage presentation of the disease. While new chemotherapy regimens, such as FOLFIRINOX (fluorouracil, leucovorin, irinotecan, and oxaliplatin) and gemcitabine with nab‐paclitaxel (nanoparticle albumin‐bound paclitaxel), have provided significant but incremental increases in overall survival (increasing to 11.1 and 8.5 months, respectively) compared with standard gemcitabine chemotherapy (6.7 months for gemcitabine alone 2, 3), the need to identify more effective therapeutic interventions remains paramount.

In a small (*n* = 34) early study 4 it was noted that the integrin αvβ6 was expressed by PDAC. Integrins regulate cellular proliferation, adhesion, migration, and invasion, and their deregulated expression and signalling can promote cancer progression 5. Integrins constitute a superfamily of 24 heterodimeric cell‐surface receptors, composed of α and β subunits, which modulate cell behaviour through bi‐directional signalling between the intracellular and extracellular compartments 6. In most normal tissues, αvβ6 expression is weak or absent, but is up‐regulated *de novo* by cells undergoing tissue remodelling, including in carcinogenesis 7. Moreover, the epithelial‐specific integrin αvβ6 previously has been described as a poor prognostic marker in multiple cancers 8, 9, 10, 11. However, a report by Hezel *et al* 12 showed that antibody inhibition of αvβ6 in a transgenic PDAC mouse model (Ptf1‐Cre;LSL‐Ras^G12D/+^;p53^fl/+^) seemed to promote PDAC progression, raising doubts as to its potential as a therapeutic target. As studies in many other cancers 8, 9, 10, 11 suggested that αvβ6 promoted cancer, and as novel pancreatic cancer therapies are needed desperately, we have examined the role of αvβ6 clinically, functionally, and therapeutically in PDAC and conclude that it should be pursued as a promising target for PDAC therapy.

## Materials and methods

### Clinical samples and mRNA expression data

The human tissues used in this study were obtained under ethical permissions local to the source of materials. All clinical data were analysed according to REMARK guidelines 13. A total of 265 PDAC clinical samples, as two separate tissue microarray (TMA) slide sets, were provided by The University of Verona, Italy, and The Beatson Institute, Scotland, UK. Matched primary and metastatic PDAC samples were supplied by the Rapid Autopsy Programme, The University of Nebraska. Eight individual cohorts of mRNA expression data, totalling 491 patients, were collected from the Barts Cancer Institute Pancreatic Expression Database, ICGC PDAC cohort (Biankin), University of Glasgow (Glasgow), TCGA PDAC cohort (PAAD), as well as previously published datasets 14, 15, 16, 17.

### Cell lines and antibodies

KRas‐mutant CFPac1, Panc04.03, and Panc1 human pancreatic cancer cell lines and the human pancreatic stellate cell line (PS1 18) were grown as adherent monolayers. Cells were grown in a 100% humidified atmosphere of 8% (volume/volume) of carbon dioxide (CO_2_)/air. Cell lines were tested routinely for, and found free of, *Mycoplasma*. All cancer cell lines were grown in Roswell Park Memorial Institute (RPMI)‐1640 medium or Dulbecco's modified Eagle's media (DMEM) (PAA Laboratories, Fisher Scientific UK, Loughborough, UK) supplemented with 10% foetal calf serum (FCS) (Biosera, Labtech International, East Sussex, UK) and glutamine (Sigma‐Aldrich, Dorset, UK; 4 mm final concentration). Human pancreatic stellate (PS1) cells were grown in a 1:1 mixture of DMEM (E15‐843; PAA Laboratories) and Ham's F12 (E15‐817; PAA Laboratories) supplemented with 10% FCS, glutamine (4 mm final concentration), and 1 μg/ml puromycin (P9620; Sigma), as a selection agent. The genetic identity of all human lines was confirmed by STR profiling (LGC Standards, Teddington, Middlesex, UK).

All clinical samples were labelled with mAb 6.2G2 (anti‐β6; a gift from Biogen Idec, Boston, MA, USA) as previously described 8. The αvβ6‐blocking mouse antibody 10D5 (MAB2077Z; Sigma‐Aldrich) was purchased and the rat mAb 53A.2 was created by JFM at Barts Cancer Institute, London. Antibody 264RAD (αvβ6‐blocking with some αvβ8‐blocking activity 19) and IgG control for pre‐clinical studies were supplied by Oncology iMED, AstraZeneca, Cambridge, UK.

### Flow cytometry and FACS

Flow cytometry was performed as previously described 8. Briefly, 2 × 10^5^ cells were incubated with 10 μg/ml of primary antibody in suspension and incubated on ice for 45 min. Controls included unstained cells and class‐matched IgG primary antibodies. The appropriate secondary AlexaFLUOR (Molecular Probes, Thermo Fisher, Loughborough, UK) antibodies were added at 1:250 and incubated for 30 min at 4°C. For flow cytometry, 10 000 events were acquired by flow cytometry on a FACS Calibur cytometer with Cell Quest Pro software version 4.0.2 (BD Biosciences, San José, CA, USA).

### *In vitro* cell proliferation assays

The optimised number of cells (3000–5000 depending on cell line) were seeded per well, in quadruplicate wells, of 96‐well plates and the following day, αvβ6 function blocking antibodies or control antibody were added at 0, 0.2, 1, 5, and 10 μg/ml final concentration. After 4 days at 37°C, fresh antibody was added to the cells at the same concentration and left until day 7. On day 7, the medium was replaced with 100 μl of MTT reagent (M5655; Sigma‐Aldrich) and generation of formazan product was quantified according to the manufacturer's instructions. Experiments were conducted with four replicates and repeated at least three times.

### Transwell® migration and invasion assays

For Transwell® assays, 5 × 10^4^ cells were seeded per well post‐treatment into 6.5 mm diameter, 8 μm pore‐size Transwells® (Corning BV, Thermo Fisher). For migration assays, the underside of the wells was coated with fibronectin (Sigma‐Aldrich) at 10 μg/ml or TGFβ1 LAP (Sigma‐Aldrich) at 1 μg/ml final concentration. For invasion assays, the upper surface of the wells was coated with 70 μl of BD Matrigel Basement Membrane matrix (Matrigel™; BD Biosciences):media (1:2 ratio). After 16 h (migration) or 72 h (invasion), cells that had migrated/invaded were trypsin/EDTA harvested and counted using an automated cell counter CASY (Scharfe Systems, Midland, Ontario, Canada) as published previously 20.

### Immunohistochemistry

Harvested tumours from mice were formalin‐fixed and processed to paraffin wax. Sections were cut at 5 μm thickness, dewaxed, and endogenous peroxidases were blocked with a 0.45% solution of H_2_O_2_ in methanol for 15 min. Antibodies to Ki67 (ab92742; Abcam, Cambridge, UK; 1:200 final dilution), cleaved caspase‐3 (9664S; Cell Signaling Technology, London, UK; 1:100 final dilution), phospho‐ERK (4376S; Cell Signaling Technology; 1:100 final dilution), endomucin (sc‐65495; Santa Cruz Biotechnology, Dallas, TX, USA; 1:200 final dilution), cytokeratin (ZO622; Dako, Agilent Technologies, Stockport, UK; 1:500 final dilution), α‐SMA (MO851; Dako; 1:300 final dilution), phospho‐Smad3 (ab52903; Abcam; 1:100 final dilution), and Smad4 (sc‐7966; Santa Cruz Biotechnology; 1:300 final dilution) were used to immunostain tumours using a standard avidin–biotin complex technique (Vectastain Elite ABC Kit; Vector Laboratories, Peterborough, UK). Picro‐Sirius red (CI 35782, Sigma‐Aldrich) was used to assess collagen deposition in tumours. Slides were scanned (Pannoramic Digital Slide Scanner, 3DHISTECH Ltd, Budapest, Hungary) and tumour staining was analysed on the Pannoramic Viewer software (version 1.15.2; 3DHISTECH Ltd) using the NuclearQuant module for Ki67, Smad4, and pErk, and the DensitoQuant module for cleaved caspase‐3, cytokeratin, and phospho‐Smad3. The number of endomucin‐positive blood vessels was counted using the Ariol ‘Angiosight’ Image Analysis module (Ariol SL‐8; Leica Microsystems, Wetzlar, Germany), and NIH ImageJ freeware (https://imagej.nih.gov/ij/download.html) was used to quantify collagen deposition and α‐SMA expression. Three independent observers performed blind scoring of the multiple cores for each cancer using the following scoring system: αvβ6 intensity staining was scored out of 4 (0: absent; 1: background staining; 2: weak; 3: moderate; 4: strong); and the percentage of epithelial cells staining positively was scored out of 4 (1: < 25%; 2: 25–50%; 3: 51–75%; 4: 76–100%), where the combined score gave a score in the range of 0–8. Median scores were determined and used for statistical analyses. We noted that the Beatson TMA stained much more weakly than the Verona TMA, possibly suggesting some degradation of the target antigen. Patients with a score greater than or equal to the median score of 3 were considered positive for αvβ6; 5 or greater was considered strong and the remaining patients with a median score of less than 3 were considered negative.

### Pre‐clinical animal studies

All animal experiments followed UK Home Office Guidelines determined by the Animals (Scientific Procedures) Act 1986. For human xenograft model development, 8‐week‐old female CD1 nu/nu mice (Charles River Laboratories, Harlow, Essex, UK) were inoculated subcutaneously (200 μl in the flank) or orthotopically (40 μl into the tail of the pancreas) with either 1 × 10^6^ CFPac1 alone or 1 × 10^6^ CFPac1 in combination with 2 × 10^6^ PS1 cells in PBS. Orthotopic xenograft tumours were harvested 6 weeks post‐injection, while subcutaneous tumours were harvested 4 weeks post‐injection. For the xenograft antibody therapy study, 8‐week‐old female CD1 nu/nu mice were obtained from Charles River Laboratories, and 1 × 10^6^ CFPac1 in combination with 2 × 10^6^ PS1 cells were injected subcutaneously. Tumours were measured with callipers bi‐weekly in two directions, where volume was calculated using the formula (width^2^ × length)/2. When tumours reached 100 mm^3^, mice were randomised to receive 4 weeks of bi‐weekly intraperitoneal injections (200 μl) of either 10 mg/kg human IgG or 264RAD, gemcitabine (Gemzar) at 100 mg/kg, or a combination of 100 mg/kg gemcitabine and 10 mg/kg 264RAD (*n* = 9 per treatment). Transgenic KDC (PdxCre^+^KRas^LSL‐G12D/+^dusp6^−/−^; described in the supplementary material, Figure S3) mouse studies were performed at the CRUK Beatson Institute, Glasgow, UK. To recapitulate the clinical setting, KDC animals showing signs of sickness (tented stance, palpable tumours) received bi‐weekly intraperitoneal injections (200 μl) of either 10 mg/kg human IgG plus 100 mg/kg gemcitabine, or 10 mg/kg 264RAD plus 100 mg/kg gemcitabine. Mice were culled when signs of severe sickness were evident. Mice were sacrificed before tumours reached Home Office volume limits, at signs of sickness, or after 6 weeks of therapy.

### Statistical analysis

The mRNA abundance data were pre‐processed as outlined previously 21. In brief, each cohort was normalised and pre‐processed independently. Pair‐wise differences between antibody‐treated versus control *in vitro* assays and immunohistochemical analysis of tumours were tested using Student's *t*‐test. Treatment differences between *in vivo* tumour growth treatments were tested using a Wald test from a normal linear mixed model fitted by maximum likelihood 22. A proportional hazards model was used to compare *in vivo* survival, with a log‐rank (score) tested for an overall difference and a Wald test between groups. Analysis was performed in the statistical software Prism GraphPad (Version 5.0b; GraphPad Software, San Diego, CA, USA) and R (R Development Core Team, 2010 2.15.1) with the nmle package. A *P* value equal to or less than 5% was considered statistically significant.

## Results

### High β6 mRNA levels correlate with poor overall survival in PDAC

**Figure 1:**
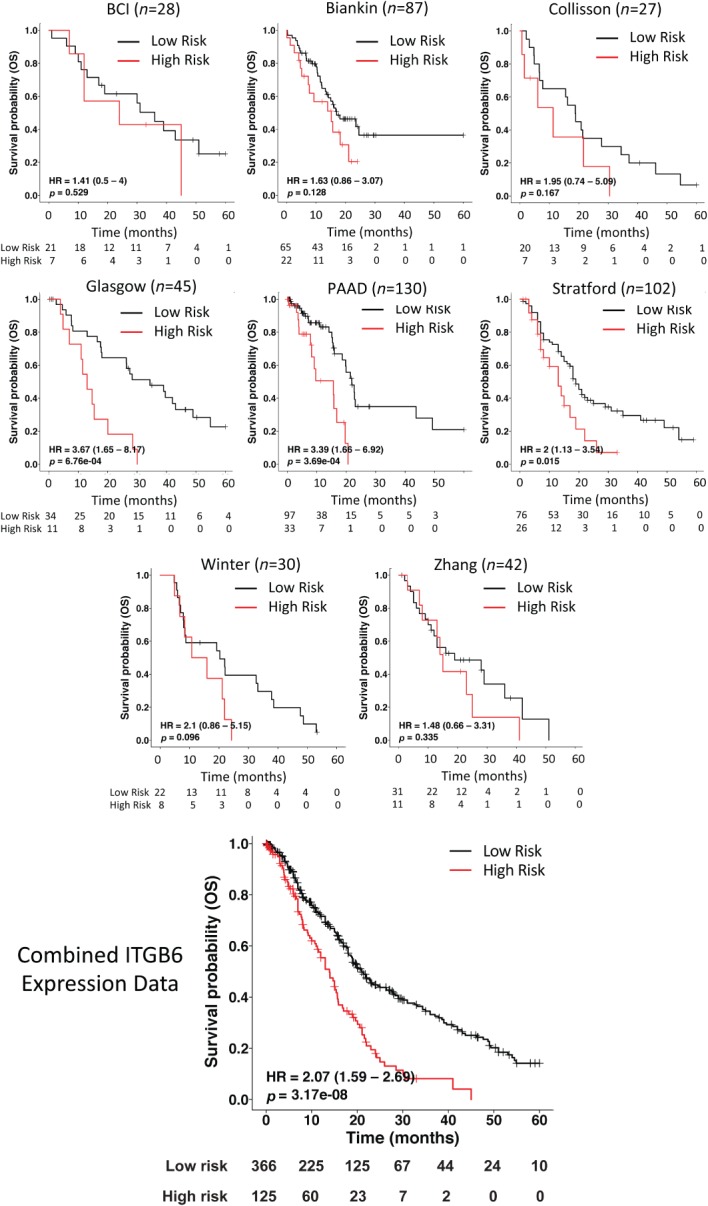
Expression of αvβ6 at the mRNA level in human PDAC. Kaplan–Meier survival curves of PDAC patients based on integrin β6 gene (*ITGB6*) expression at the mRNA level from eight cohorts: Barts Cancer Institute (BCI) pancreatic expression database, IGCG PDAC cohort (Biankin), University of Glasgow (Glasgow), TCGA PDAC cohort (PAAD), as well as previously published datasets (Collisson, Stratford, Winter, and Zhang 14, 15, 16, 17). The red line indicates the upper quartile of *ITGB6*‐positive patients and the black line indicates *ITGB6*‐weakly positive or ‐negative patients. The lower histogram shows the combined Kaplan–Meier survival analysis of the total of 491 PDAC patients based on integrin β6 mRNA (*ITGB6*) expression. Again, the red line indicates the upper quartile of *ITGB6*‐positive patients (*n* = 125) and the black line *ITGB6*‐weakly positive or ‐negative patients (*n* = 366). Hazard ratio (HR) = 2.07 (95% CI 1.59–2.69); log‐rank test *p* = 3.17 × 10^−8^.

Eight independent cohorts of PDAC gene expression datasets, totalling 491 PDAC cases, were each dichotomised into high (top 25%; red line, Figure 1) and low (bottom 75%; black line, Figure 1) expressers based on *ITGB6* mRNA expression levels (mRNA abundance for each cohort is recorded in the supplementary material, Table S1). Combined data show that with high expressers of *ITGB6* (red line; *n* = 125), there was a significantly lower overall survival compared with weak expressers of *ITGB6* (black line; *n* = 366) [hazard ratio (HR): 2.07; 95% confidence interval (CI): 1.59–2.69; log‐rank test: *p* = 3.17 × 10^−8^] (Figure 1, lower histogram).

### αvβ6 protein is expressed by most PDACs and is retained by metastases

**Figure 2:**
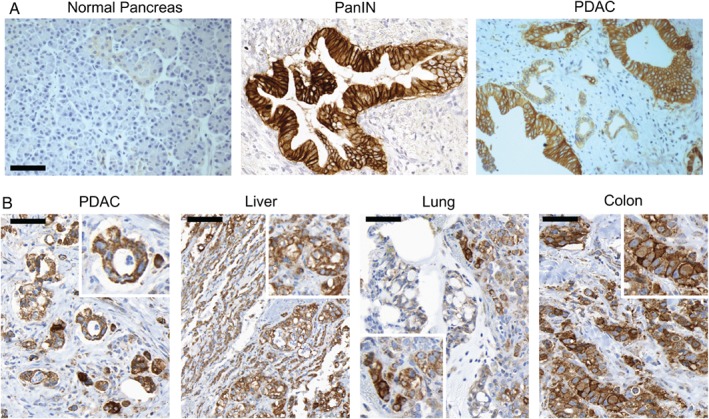
Expression of αvβ6 protein in human PDAC. FFPE PDAC tissues were labelled with mAb 6.2G2 to assess αvβ6 expression. (A) Representative images of human normal pancreas, PanIN, and invasive PDAC tumour tissue stained for αvβ6 expression are shown. Scale bar = 100 μm. (B) Representative staining for αvβ6 expression in a primary tumour and paired metastases from a single patient is shown, with an enlarged inset for improved clarity. Scale bar = 50 μm.

Two separate cohorts of PDAC samples were analysed independently for expression of αvβ6. The Beatson cohort had 118 PDAC cases and 12 normal pancreata, whereas the Verona dataset had 147 patients and 20 normal pancreata. Examples of typical staining are shown in Figure 2A. Expression of αvβ6 was restricted specifically to epithelial cells as observed previously 7, 8. Normal pancreas samples were mostly negative for αvβ6, though occasionally some duct cells expressed αvβ6 weakly. Expression of αvβ6 was detected in 98% (median score ≥ 3) of Verona samples and 83% of Beatson samples, although staining intensity was uniformly weaker on the Beatson TMA and thus staining may be an underestimate. Using a median score of ≥ 5 to represent strong staining (as shown on the PanIN and PDAC in Figure 2A), 84% of Verona PDAC samples expressed αvβ6 strongly. We then examined six PDAC patients for whom we had primary tumour tissue and their matched metastatic tumours from lung, colon or liver; all the primary tumours expressed αvβ6 strongly and all metastases were also αvβ6‐positive, 70% retaining similarly strong expression to that of the matched primary (Figure 2B and Table 1).

**Table 1:** Expression of αvβ6 in primary PDAC and matched metastases

	Tumour site	Cases	Strong αvβ6 expression	Moderate, weak, nil αvβ6 expression
Primary	Pancreas	6	6 (100%)	0 (0%)
Metastases	Liver	6	3 (50%)	3 (50%)
	Lung	2	2 (100%)	0 (0%)
	Colon	5	4 (80%)	1 (20%)

Sections of primary PDAC tumours and patient‐matched secondary tumours were collected from six patients (generous gift from the Rapid Autopsy Programme, The University of Nebraska) and stained for expression of αvβ6 using immunohistochemistry. All primary tumours expressed αvβ6 strongly (median score ≥ 5) and all metastases retained αvβ6 expression, 70% retaining strong expression.

### Blockade of αvβ6 reduces proliferation, migration, and invasion of αvβ6‐positive pancreatic cancer cells *in vitro*

**Figure 3:**
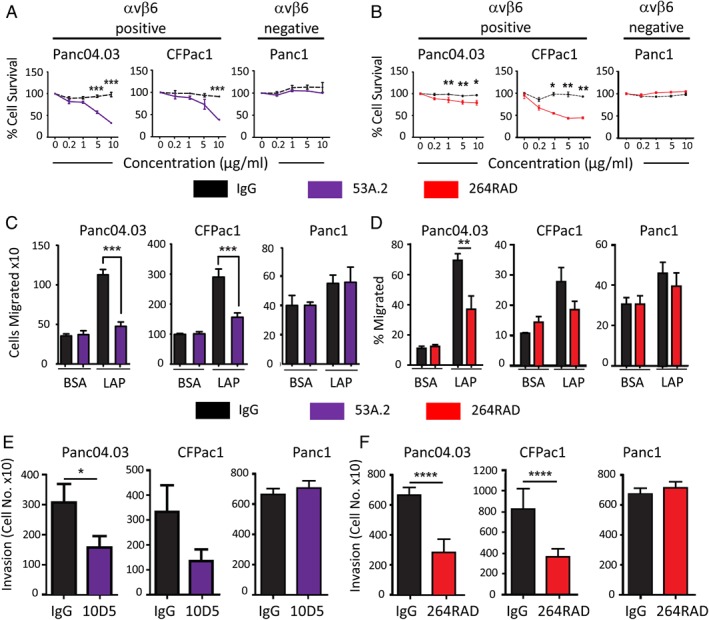
Antibody blockade of αvβ6 inhibits proliferation, migration, and invasion of PDAC cell lines. Proliferation of Panc04.03, CFPac1, and Panc1 cells treated with varying concentrations of αvβ6 function‐blocking antibodies, (A) 53A.2 (purple) or (B) 264RAD (red), versus IgG control (black). Two‐way ANOVA (Bonferroni's correction), *p < 0.05; **p < 0.01; ***p < 0.001. Migration of PDAC cells towards the latency‐associated peptide (LAP) of TGFβ, compared with BSA control, following incubation with (C) 53A.2 (purple) or (D) 264RAD (red) versus IgG control (black) used at the same concentration. Data show the mean ± SEM of three independent experiments performed in quadruplicates. Two‐way ANOVA, **p < 0.005; ***p < 0.001. Invasion of PDAC cells through Matrigel™ following treatment with (E) 10D5 (purple) or (F) 264RAD (red) compared with IgG control antibody (black) used at the same concentration. The number of invaded cells was counted after 72 h. Data show the mean ± SEM of three independent experiments performed in triplicate. Student's *t*‐test, *p < 0.05; ****p < 0.0001.

Integrin αvβ6 was expressed on seven out of nine pancreatic tumour cell lines, as determined by flow cytometry (see supplementary material, Table S2). The αvβ6‐blocking antibodies 53A.2 (Figure 3A) and 264RAD (Figure 3B) similarly and significantly reduced the proliferation of αvβ6‐positive, but not αvβ6‐negative, PDAC cancer cells. Both antibodies also reduced the migration of αvβ6‐positive Panc04.03 and CFPac1 pancreatic cancer cells towards their ligand LAP (Figure 2C,D). 10D5 (αvβ6‐blocking) and 264RAD also reduced the invasion of these αvβ6‐positive cells through Matrigel™‐coated Transwells® (Figure 3E,F), compared with control‐treated cells and αvβ6‐negative Panc1 cells. Thus, αvβ6 promotes PDAC cancer cell growth, migration, and invasion.

### 264RAD antibody therapy reduces the growth of pancreatic human xenograft tumours

CFPac1 cells were injected alone or in combination with a human pancreatic stellate cell (PSC) line (PS1) orthotopically or subcutaneously. The presence of PSCs reduced the number and density of endomucin‐positive blood vessels and increased collagen deposition compared with CFPac1 cells growing alone (see supplementary material, Figure S1). Since both the subcutaneous and the orthotopic tumours with PSCs developed similar hypovascular and desmoplastic stroma seen in human pancreatic cancer 23, 24, we used the more easily tractable subcutaneous xenograft model for the αvβ6‐blocking 264RAD 19 antibody therapy studies *in vivo*.

**Figure 4:**
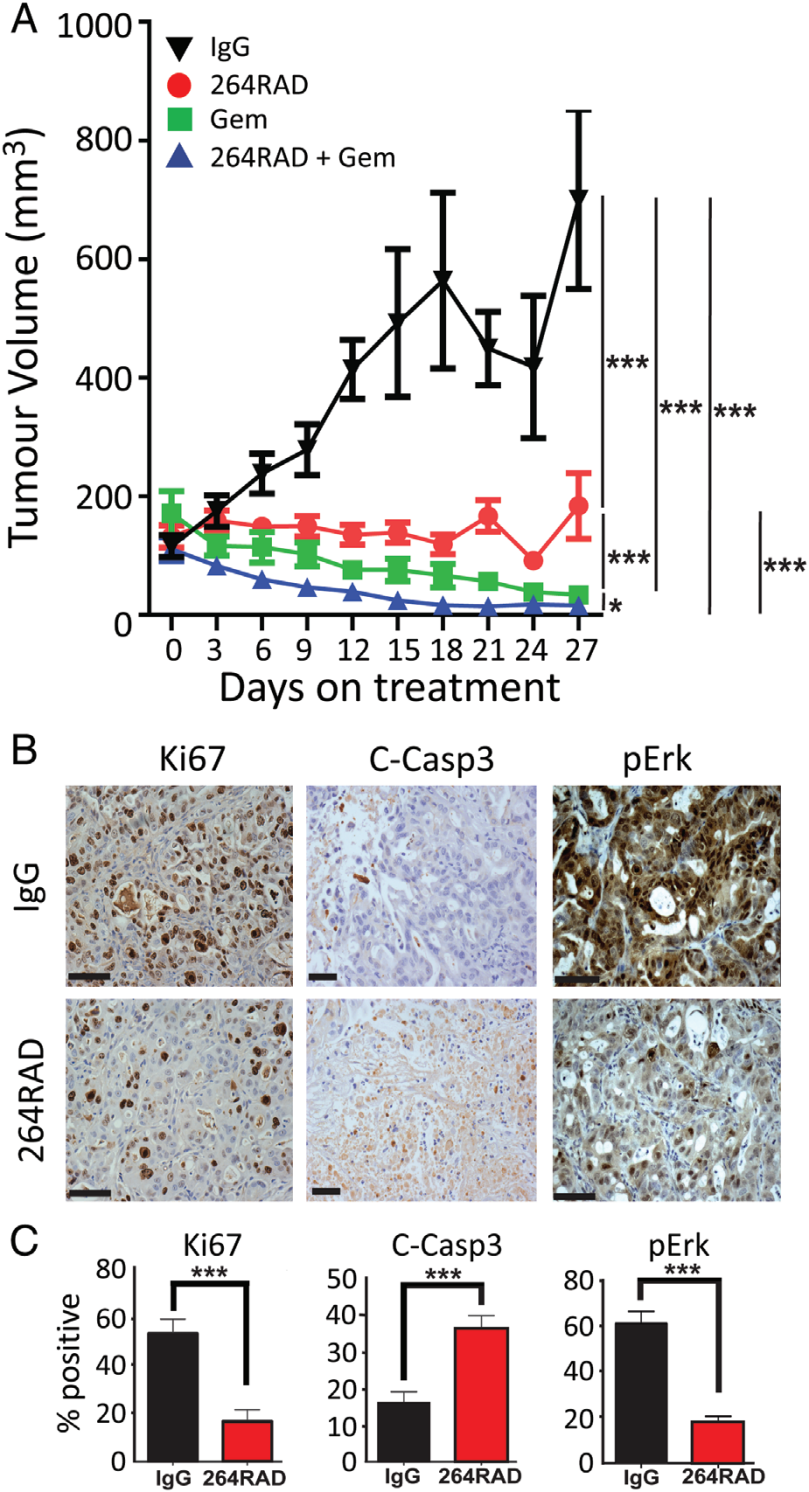
264RAD therapy in the CFPac1/PS1 subcutaneous human xenograft mouse model of pancreatic cancer. (A) A total of 0.5 × 10^6^ CFPac1 cells and 1 × 10^6^ PS1 cells were co‐injected subcutaneously into female CD1nu/nu mice. When tumours reached approximately 100 mm^3^, mice were entered into four different treatment arms: IgG isotype control antibody (10 mg/kg); 264RAD (10 mg/kg); gemcitabine (100 mg/kg); or 264RAD (10 mg/kg) and gemcitabine (100 mg/kg) (*n* = 9 per group). Animals were treated bi‐weekly for 4 weeks. Data were analysed using a linear mixed model fitted to log volume 22; *p < 0.05; ***p < 0.0001. (B) Representative images and (C) quantification of IgG‐treated and 264RAD‐treated tumours stained for Ki67, cleaved caspase‐3, and phosphorylated ERK (pERK). Student's *t*‐test, ***p < 0.0001. Scale bar = 50 μm.

Treatment of mice bearing 100 mm^3^ CFPac1/PS1 subcutaneous tumours with 264RAD demonstrated significantly reduced tumour growth, compared with isotype control (*p* ≤ 0.0001) (Figure 4A). Treatment of tumours with gemcitabine alone also significantly reduced tumour growth compared with isotype control (*p* ≤ 0.0001), or when compared with 264RAD monotherapy (*p* = 0.012). However, the combination of 264RAD with gemcitabine demonstrated the greatest reduction in tumour volume of all mice during treatment, compared with isotype control (*p* ≤ 0.0001), or compared with gemcitabine alone (*p* = 0.028) (Figure 4A and see supplementary material, Figure S2). In fact, the tumours in three mice treated with 264RAD plus gemcitabine vanished completely (see supplementary material, Figure S2). No toxicity (loss of weight, change in appearance or behaviour) was observed in any mice during any treatment (data not shown).

To determine the molecular mechanisms associated with 264RAD‐induced tumour reduction, immunochemistry demonstrated a significant down‐regulation of proliferation (Ki67) and growth signalling (phosphorylated Erk), and a significant up‐regulation of apoptosis (cleaved caspase‐3; Figure 4B) in the 264RAD‐treated tumours compared with control‐treated tumours (*p* ≤ 0.0001) (Figure 4C).

### Therapy with the αvβ6‐blocking 264RAD antibody significantly improves survival in immunocompetent transgenic mice bearing αvβ6‐expressing PDAC tumours

**Figure 5:**
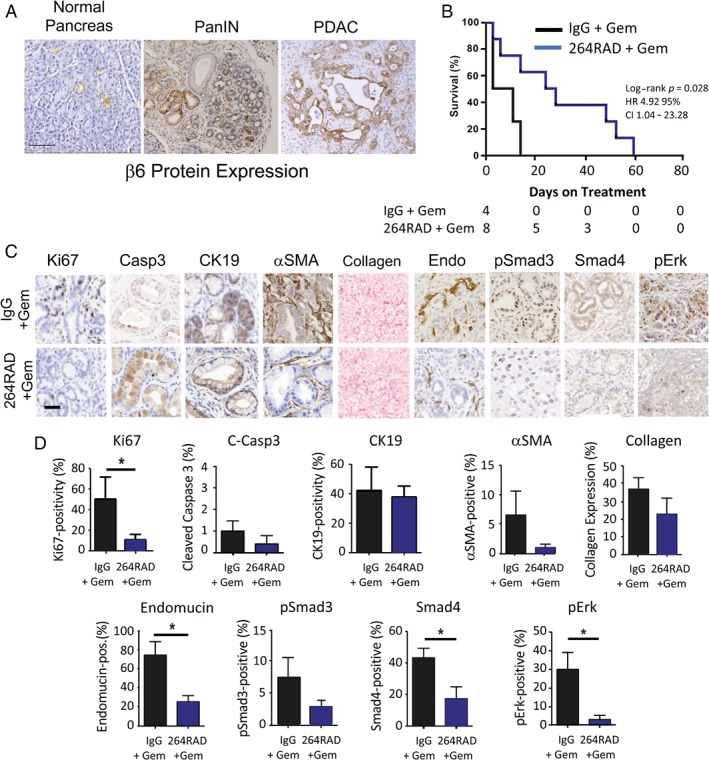
Treatment of PDAC‐bearing KDC mice with 264RAD and immunohistochemical analysis. (A) Representative immunohistochemical analysis of αvβ6 expression in normal, PanIN, and PDAC from PdxCre^+^KRas^LSL‐G12D/+^Dusp6^−/−^ (KDC) mice. Normal pancreas shows no αvβ6 on acinar cells and weak staining on some ductal cells. Strong expression of αvβ6 protein was evident in PanIN and PDAC. (B) Kaplan–Meier survival curve of KDC mice treated with IgG isotype control (10 mg/kg) and gemcitabine (100 mg/kg) (grey line; *n* = 4), or 264RAD (10 mg/kg) and gemcitabine (100 mg/kg) (purple line; *n* = 8). A significant increase in survival upon treatment with 264RAD is evident. Hazard ratio (HR) = 4.92 (CI: 1.04–23.28); log‐rank *p* = 0.028. (C) PDAC tumours from KD mice treated with IgG isotype control and gemcitabine or 264RAD and gemcitabine were analysed using immunohistochemistry for markers of tumourigenesis. Representative images of staining for Ki67, cleaved caspase‐3 (C‐Casp‐3), cytokeratin 19 (CK19), alpha‐smooth muscle actin (αSMA), collagen, endomucin Endo), phosphorylated Smad3 (pSmad3), nuclear Smad4, and phosphorylated Erk (pErk) are displayed. Scale bar = 50 μm. (D) Quantification of staining using image analysis as described in the Materials and methods section. Data show the mean ± SEM of three tissue samples per group. Student's *t*‐test, *p = 0.05.

We sought to determine the effect of 264RAD antibody therapy on PDAC using immunocompetent transgenic mice. The pathological development of PDAC in well‐established KPC (PdxCre^+^KRas^LSL‐G12D/+^p53^LSL‐R172H/+^) mice closely matches the progression of human disease 25. However, unlike in humans where αvβ6 is expressed on most human PDACs (see above), integrin αvβ6 was detected only in PanIN lesions but not in the PDAC primary tumours of KPC mice (data not shown). Therefore, we examined a new transgenic pancreatic cancer model called KDC (PdxCre^+^KRas^LSL‐G12D/+^dusp6^−/−^), whose mice possess the same activating mutation in KRas as KPC mice together with deletion of the dual‐specificity phosphatase 6 (dusp6) targeted to the pancreas. These mice develop PanIN lesions which progress to invasive, metastatic PDAC between 3 and 10 months of age (see supplementary material, Figure S3). Similar to human disease, we discovered that KDC mice demonstrated a striking expression of αvβ6 in PanINs and primary tumours (Figure 5A). Mirroring human therapy, in which therapeutic intervention is usually administered in late‐stage disease, KDC mice with palpable pancreatic tumours and displaying visible symptoms of sickness were treated with gemcitabine in combination with 264RAD or isotype control antibody. The 264RAD‐treated mice showed significantly increased overall survival in comparison with isotype control treated animals (log‐rank test *p* = 0.028; HR: 4.92; 95% CI: 1.04–23.28) (Figure 5B). Immunohistochemistry revealed that 264RAD treatment of PDAC‐bearing KDC mice significantly reduced tumour cell proliferation (Ki67), tumour growth signalling (pErk), blood vessel density (endomucin), and TGFβ signalling (nuclear Smad4), and showed a trend towards reduced phosphorylated Smad3, αSMA, and collagen deposition (Figure 5C,D).

## Discussion

Cytotoxic pharmaceuticals remain the principal tool for therapy of PDAC and while there have been promising developments that offer incremental increases in survival of additional months 2, 3 patients still need vastly improved therapies if we are to significantly improve upon the 5‐year survival rate of less than 4%. Our data suggest that combining blockade of integrin αvβ6 with cytotoxic therapy could significantly help to achieve that goal.

We, and others, have reported that high expression of the pro‐invasive integrin αvβ6 correlates with poor overall survival from cancer 8, 9, 10, 11. Thus, when Sipos *et al* reported 4 that all of their 34 PDAC cases analysed expressed αvβ6, it was reasonable to assume that this integrin would also promote this disease. However, a robust study from Hezel *et al* 12 suggested that αvβ6 was a tumour suppressor in PDAC, probably through local activation of TGFβ. It is well established that TGFβ can be both tumour‐suppressive and tumour‐promoting (reviewed in refs 26 and 27). A major mechanism by which TGFβ suppresses normal epithelial growth is through activation of cyclin‐dependent kinases p21 and p15. However, carcinoma cells often develop mutations that make them refractory to this growth suppression and allowing them to respond directly to the ability of TGFβ to promote invasion or indirectly by TGFβ‐dependent generation of a tumour‐permissive microenvironment 26. Hezel *et al* used the Ptf1‐Cre;LSL‐Ras^G12D/+^;p53^fl/+^ mice that develop PDAC relatively early, at 9–10 weeks 12. They reported that antibody blockade of αvβ6 (or TGFβ) promoted early development of PanINs and progression to PDAC, but had no effect on the tumour microenvironment, and that these observations correlated with TGFβ signalling and αvβ6 expression. Thus, their data are consistent with the conclusion that the transformed pancreatic ductal cells in Ptf1‐Cre;LSL‐Ras^G12D/+^;p53^fl/+^ mice retain the TGFβ‐dependent growth‐suppressive signalling pathways even when they have developed into PanINs and PDAC. Another mechanism that may be relevant was reported by David *et al* 28 who showed that PDAC cells that retained functional SMAD4 signalling underwent TGFβ‐dependent EMT that transitioned to apoptosis driven by the repurposing of the transcription factor Sox4. Thus, in this situation, inhibition of αvβ6‐dependent activation of TGFβ would reduce apoptosis and enhance PDAC survival. However, the number of PDAC tumours retaining TGFβ‐dependent SMAD signalling is likely to be a smaller fraction of humans with PDAC, since most human PDACs have genetic dysfunction in TGFβ signalling 26, 27.

Analysis of the expression of *ITGB6* mRNA on eight separate cohorts showed that high (top 25% quartile) expression, in general, correlated with poorer survival. When the data from all 491 PDAC cases were analysed, the high expression group was associated with significantly poorer survival (Figure 1; HR: 2.07; log‐rank test: *p* = 3.17 × 10^−8^). Thus, there is a clear association linking β6 and survival from PDAC. Our data also showed that in addition to most PDACs expressing αvβ6 on their surface, the integrin is retained by metastases, so therapies directed to this integrin would target both primary and secondary disease.

To investigate the role of αvβ6 in PDAC experimentally, we examined a panel of PDAC cell lines and confirmed that seven of nine expressed αvβ6 (see supplementary material, Table S2). When we grew αvβ6‐expressing (Panc0403 and CFPac1) and αvβ6‐negative (Panc1) cell lines in the presence of two different αvβ6‐blocking antibodies (53a2 and 264RAD), we noted that, compared with control IgG, both αvβ6‐blocking antibodies suppressed growth (Figure 3A,B). This is consistent with the report of Singh *et al* 29 that the *ITGB6* gene is required for *Ras*‐oncogene‐dependent growth of PDAC cells. We further showed that αvβ6 promoted migration and invasion (Figure 3C–F), confirming that, as has been shown for other cell types 8, 20, 30, αvβ6 imparts greater proliferative, migratory, and invasive capacity to PDAC cells.

To establish whether αvβ6 could be an effective therapeutic target, we tested two novel PDAC tumour models that reflect human disease. We generated a xenograft from combined injection of human PDAC and pancreatic stellate cells (CFPac1/PS1) that developed desmoplastic, hypoangiogenic tumours, mimicking human disease 23. Treatment with 264RAD, which blocks αvβ6 function, stopped established tumours growing and enhanced gemcitabine therapy (*p* < 0.0001), even curing 33% of mice in the combination therapy group (see supplementary material, Figure S3). Mechanistically, the 264RAD antibody increased apoptosis and blocked pErk growth signals and tumour cell division (Figure 4C). Thus, just as we have reported in breast and oral tumours 8, 19 inhibition of αvβ6 with 264RAD could abrogate and sometimes even eliminate established PDAC tumours.

**Figure 6:**
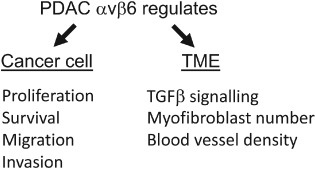
Mechanisms by which integrin αvβ6 promotes pancreatic ductal adenocarcinoma. Our data show that αvβ6 promotes PDAC by modulating the behaviour of both the cancer cells and the tumour microenvironment.

The human xenograft studies required immunodeficient mice and thus we turned to a new transgenic PDAC mouse model, KDC (PdxCre^+^KRas^LSL‐G12D/+^dusp6^−/−^), which combines a *Ras* ^*G12D*^ mutation with loss of dual‐specific phosphatase 6 (Dusp6), considered a potential tumour suppressor in PDAC 31; in KDC mice, αvβ6 was expressed in PanINs and PDAC tumours (Figure 5). Blockade of αvβ6 with 264RAD in combination with gemcitabine significantly (HR: 4.92; log‐rank test *p* = 0.028) increased survival of KDC mice (Figure 5). This is in agreement with our human xenograft study but is in contrast to the report by Hezel *et al* that suggested a tumour‐suppressive role of αvβ6 in PDAC 12. Moreover, immunohistochemistry results revealed that tumours from KDC mice treated with 264RAD had significantly reduced proliferation (Ki67), tumour growth signalling (pErk), blood vessel density (endomucin), and TGFβ signalling (nuclear Smad4), and showed a trend towards reduced desmoplasia (reduced αSMA‐expressing myofibroblasts and collagen deposition). Thus, antibody blockade of αvβ6 correlated with both direct anti‐tumour cell effects (reduced pErk, Ki67) and generation of a less tumour‐permissive stroma. Together with reduced migratory and invasive capacity, these effects combine to suppress PDAC growth and increase overall survival. Figure 6 shows our current model of how αvβ6 affects PDAC growth.

Of all cancer conditions, PDAC remains the one that is most desperate for novel therapeutic strategies. As αvβ6 is expressed by the majority of human PDACs, and as our clinical, functional, and pre‐clinical animal studies combine to show that this integrin actively promotes this disease, we must consider αvβ6 as a promising target for the therapy of PDAC in combination with conventional therapies.

## Author contributions statement

JFM conceived and directed the study, and wrote the final manuscript. All the authors critically assessed, edited, and approved the final manuscript. JFM, HMK, OJS, and STB supervised and coordinated the study, and analysed the data. SV, CSR, CWS, AD, and KMM performed the biological experiments, collected and analysed data, and contributed to writing. TRJE, OJS, JPM, SK, and CSR generated, supervised, and analysed the data from the KDC transgenic studies. PB, DC, NBJ, AB, and CC generated databases used in the study. SH, AB, CC, and JPM performed statistical analyses on clinical and pre‐clinical data. AS and RL collected and supplied clinical material and associated clinical data for immunohistochemistry studies on Verona samples. SK generated the KDC mouse.

**SUPPLEMENTARY MATERIAL ONLINE**

**Supplementary figure legends**

**Figure S1.** Generation and validation of a human PDAC tumour‐mimetic using αvβ6‐positive PDAC cells combined with pancreatic stellate cells

**Figure S2.** Survival and individual tumour growth curves of antibody‐treated CFPac1/PS1 subcutaneous xenograft mouse models of pancreatic cancer

**Figure S3.** Characterisation of pancreatic cancer progression in KDC mice

**Table S1.** Distribution of *ITGB6* log_2_ mRNA abundance across the clinical cohorts

**Table S2.** Integrin screening of a panel of PDAC cell lines by flow cytometry.

## Supporting information


**Figure S1.** Generation and validation of a human PDAC tumour‐mimetic using αvβ6‐positive PDAC cells combined with pancreatic stellate cells

**Figure S2.** Survival and individual tumour growth curves of antibody‐treated CFPac1/PS1 subcutaneous xenograft mouse models of pancreatic cancer

**Figure S3.** Characterisation of pancreatic cancer progression in KDC mice

**Table S1.** Distribution of ITGB6 Log2 mRNA abundance across the clinical cohorts

**Table S2.** Integrin screening of a panel of PDAC cell lines by flow cytometry
