# Supplementary figures and images for: The integrin αvβ6 drives pancreatic cancer through diverse mechanisms and represents an effective target for therapy
Source: J Pathol. 2019 Jul 30;249(3):332–42. doi: 10.1002/path.5320 (PMC6852434; doi:10.1002/path.5320)

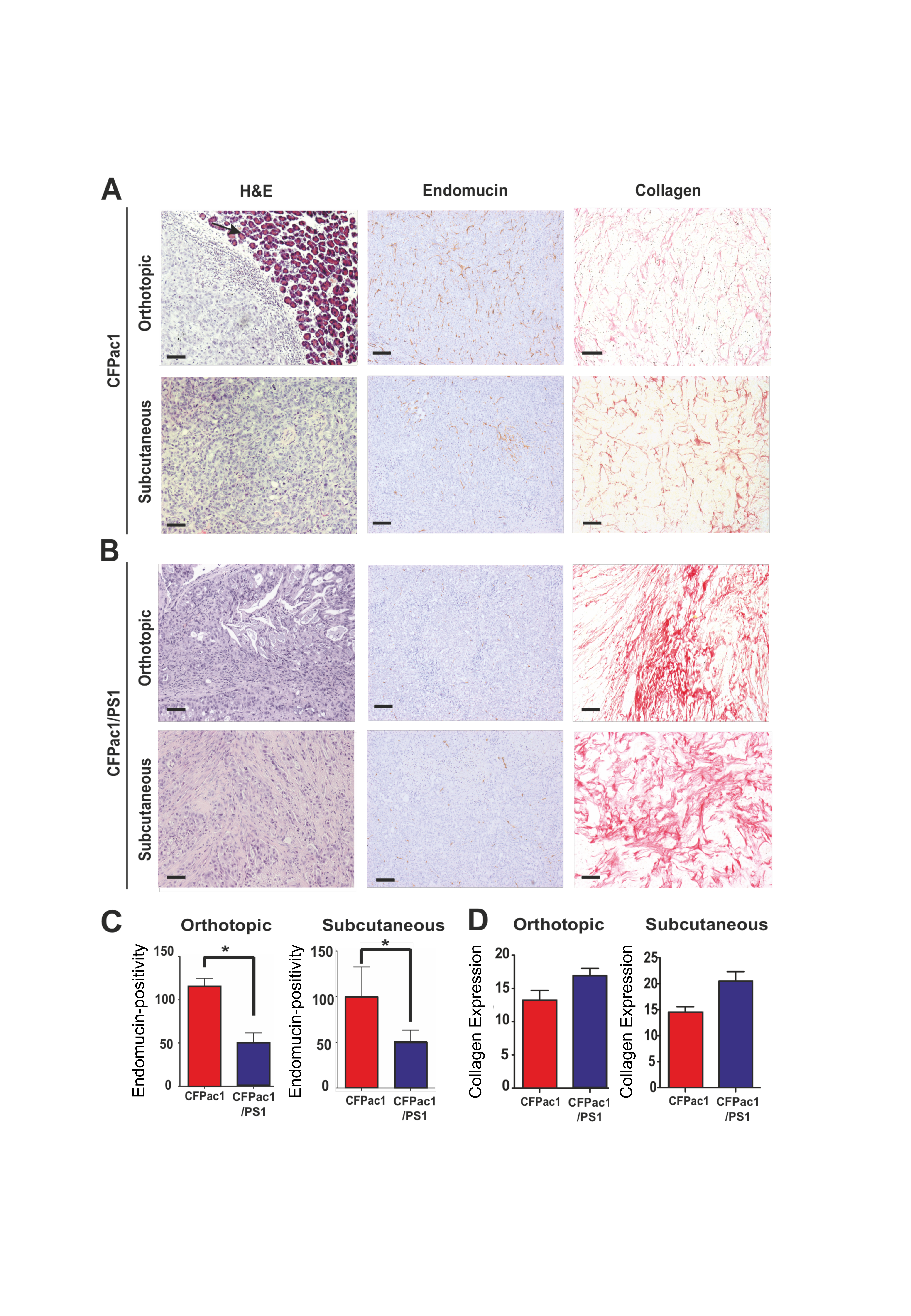

Supplement: Supplementary file 1 — Figure S1. Generation and validation of a human PDAC tumour‐mimetic using αvβ6‐positive PDAC cells combined with pancreatic stellate cells [file PATH-249-332-s001.tif]

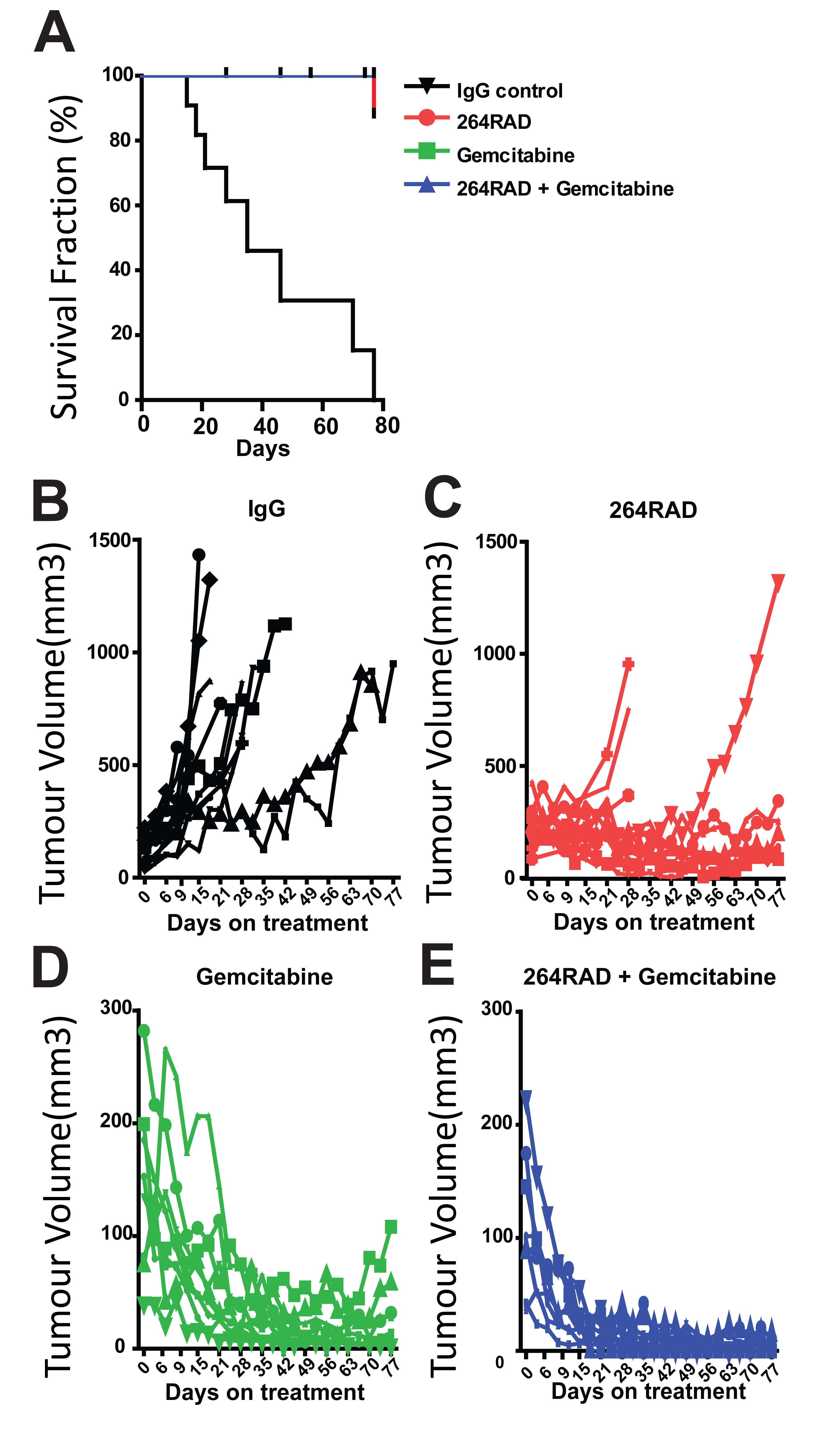

Supplement: Supplementary file 2 — Figure S2. Survival and individual tumour growth curves of antibody‐treated CFPac1/PS1 subcutaneous xenograft mouse models of pancreatic cancer [file PATH-249-332-s002.tif]

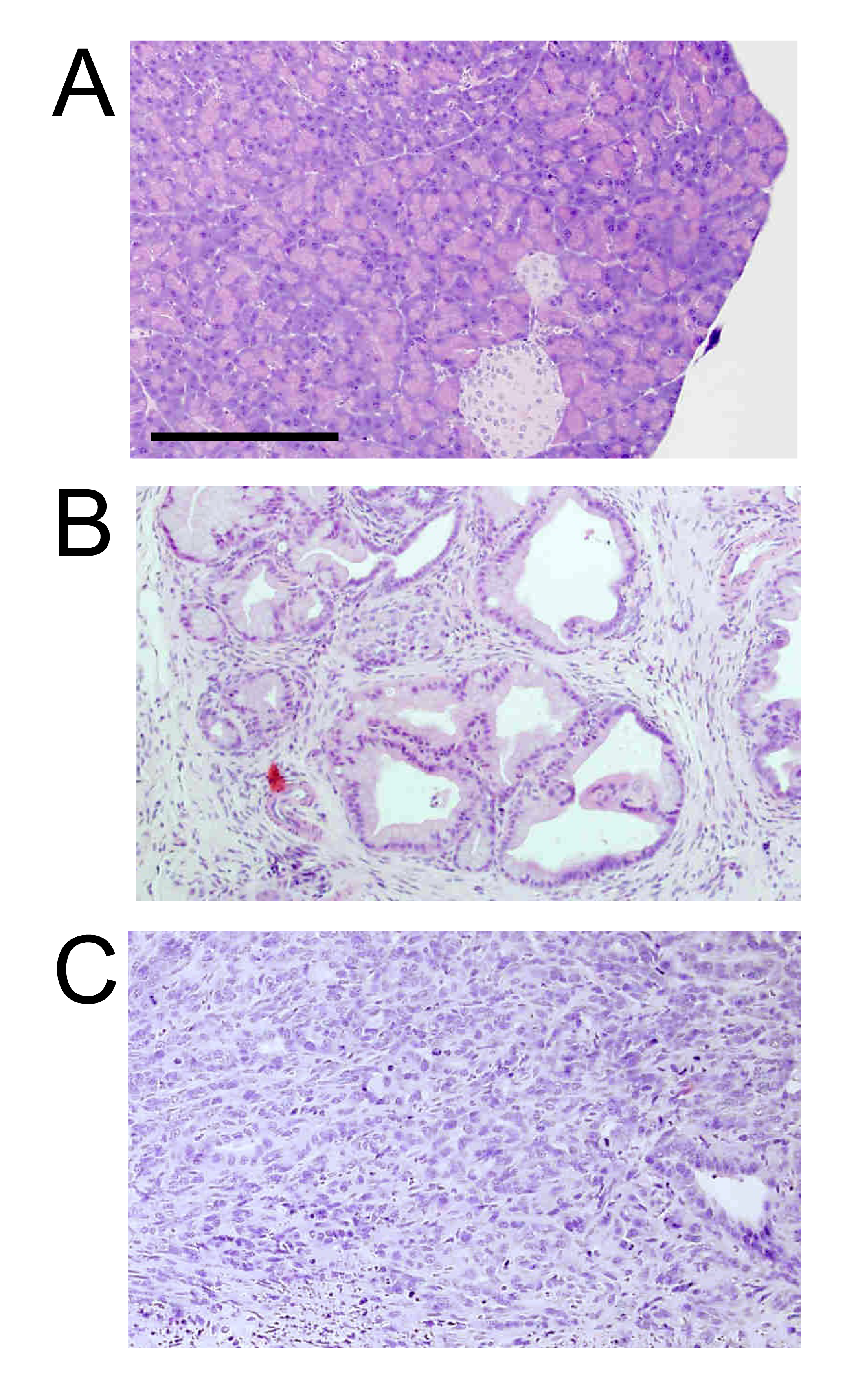

Supplement: Supplementary file 3 — Figure S3. Characterisation of pancreatic cancer progression in KDC mice [file PATH-249-332-s003.tif]
