# Supplementary material for: The integrin αvβ6 drives pancreatic cancer through diverse mechanisms and represents an effective target for therapy
Source: J Pathol. 2019 Jul 30;249(3):332–42. doi: 10.1002/path.5320 (PMC6852434; doi:10.1002/path.5320)
Supplement: Supplementary file 4 — Table S1. Distribution of ITGB6 Log2 mRNA abundance across the clinical cohorts [file PATH-249-332-s004.docx]

**The integrin αvβ6 drives pancreatic cancer through diverse mechanisms and represents an effective target for therapy**

Reader CS *et al*. *J Pathol* DOI: 10.1002/path.5320

**Table S1. Distribution of *ITGB6* log_2_ mRNA abundance across the clinical cohorts**

|  | **Min.** | **1st quartile** | **Median** | **Mean** | **3rd quartile** | **Max.** |
| --- | --- | --- | --- | --- | --- | --- |
| **BCI-All** | 6.722 | 9.908 | 10.447 | 10.208 | 10.733 | 11.77 |
| **BCI-Low** | 6.722 | 9.219 | 10.338 | 9.831 | 10.479 | 10.703 |
| **BCI-High** | 10.821 | 11.186 | 11.346 | 11.339 | 11.531 | 11.77 |
| **Biankin-All** | 7.272 | 7.465 | 7.664 | 7.711 | 7.87 | 8.531 |
| **Biankin-Low** | 7.272 | 7.448 | 7.529 | 7.577 | 7.71 | 7.867 |
| **Biankin-High** | 7.873 | 7.956 | 8.02 | 8.107 | 8.324 | 8.531 |
| **Collisson-All** | 3.43 | 5.426 | 6.173 | 6.167 | 6.912 | 8.427 |
| **Collisson-Low** | 3.43 | 5.338 | 5.638 | 5.623 | 6.303 | 6.831 |
| **Collisson-High** | 6.993 | 7.149 | 7.838 | 7.722 | 8.25 | 8.427 |
| **Stratford-All** | −0.268 | 2.475 | 3.548 | 3.449 | 4.464 | 7.03 |
| **Stratford-Low** | −0.268 | 2.141 | 3.175 | 2.817 | 3.641 | 4.447 |
| **Stratford-High** | 4.47 | 4.794 | 5.102 | 5.297 | 5.686 | 7.03 |
| **Winter-All** | 4.669 | 5.701 | 6.696 | 6.583 | 7.762 | 8.614 |
| **Winter-Low** | 4.669 | 5.285 | 5.861 | 6.01 | 6.727 | 7.637 |
| **Winter-High** | 7.804 | 7.965 | 8.113 | 8.159 | 8.346 | 8.614 |
| **Zhang-All** | 4.612 | 7.641 | 8.43 | 8.117 | 8.692 | 10.339 |
| **Zhang-Low** | 4.612 | 7.126 | 7.889 | 7.677 | 8.47 | 8.68 |
| **Zhang-High** | 8.696 | 8.94 | 9.382 | 9.357 | 9.697 | 10.339 |
| **PAAD-All** | 5.159 | 11.366 | 11.869 | 11.899 | 12.614 | 14.717 |
| **PAAD-Low** | 5.159 | 11.081 | 11.642 | 11.383 | 12.021 | 12.614 |
| **PAAD-High** | 12.614 | 12.942 | 13.334 | 13.417 | 13.808 | 14.717 |
| **Glasgow-All** | 3.03 | 6.6 | 7.94 | 7.58 | 8.68 | 10.99 |
| **Glasgow-Low** | 3.03 | 6.355 | 7.135 | 6.916 | 8.16 | 8.68 |
| **Glasgow-High** | 8.69 | 8.97 | 9.56 | 9.632 | 9.99 | 10.99 |

Relative distribution of mRNA levels is recorded in the table as log_2_ mRNA. 'All' represents distribution across all samples, while 'Low' and 'High' groups correspond to distribution across the two groups dichotomised on cohort-specific 75th percentile of mRNA abundance.
