# Supplementary material for: The integrin αvβ6 drives pancreatic cancer through diverse mechanisms and represents an effective target for therapy
Source: J Pathol. 2019 Jul 30;249(3):332–42. doi: 10.1002/path.5320 (PMC6852434; doi:10.1002/path.5320)
Supplement: Supplementary file 5 — Table S2. Integrin screening of a panel of PDAC cell lines by flow cytometry [file PATH-249-332-s005.docx]

**The integrin αvβ6 drives pancreatic cancer through diverse mechanisms and represents an effective target for therapy**

Reader CS *et al*. *J Pathol* DOI: 10.1002/path.5320

**Table S2.** Integrin screening of a panel of PDAC cell lines by flow cytometry

|  | Well differentiated | | | | | Poorly/moderately differentiated | | | |
| --- | --- | --- | --- | --- | --- | --- | --- | --- | --- |
| Cell line | **CFPac1** | **Capan1** | **Colo357** | **Panc04.03** | **HPAF** | **AsPc1** | **BxPc3** | **Panc1** | **MiaPaCa2** |
| α1 | ++ | ++ | ++ | + | − | − | + | − | − |
| α2 | +++ | ++++ | +++ | ++++ | +++ | ++++ | ++++ | +++ | − |
| α5 | + | − | − | + | − | − | + | + | + |
| αvβ3 | + | − | − | − | − | − | + | + | − |
| αvβ5 | + | + | − | ++ | − | + | − | ++ | − |
| αvβ6 | ++ | ++ | ++ | +++ | +++ | + | ++ | − | − |

A panel of nine human PDAC cell lines, which originated from well- and poorly/moderately differentiated PDAC tumours, was screened for integrin expression using flow cytometry. Average geometric mean fluorescence intensity (MFI) values from repeat experiments (*n* ≥ 3) are represented as a + to ++++ scale: 1–10 (−; no expression); 11–20 (+; weak expression); 21–50 (++; moderate expression); 51–100 (+++; high expression); > 100 (++++; very high expression). Class control MFI was always less than 10.
